# Conservation priorities and distribution patterns of vascular plant species along environmental gradients in Aberdare ranges forest

**DOI:** 10.3897/phytokeys.131.38124

**Published:** 2019-09-16

**Authors:** Solomon Kipkoech, David Kimutai Melly, Benjamin Watuma Mwema, Geoffrey Mwachala, Paul Mutuku Musili, Guangwan Hu, Qingfeng Wang

**Affiliations:** 1 CAS Key Laboratory of Plant Germplasm Enhancement and Specialty Agriculture, Wuhan Botanical Garden, Chinese Academy of Sciences, Wuhan 430074, Hubei, China East African Herbarium, National Museums of Kenya Nairobi Kenya; 2 University of Chinese Academy of Sciences. Beijing 100049, China Wuhan Botanical Garden, Chinese Academy of Sciences Wuhan China; 3 Sino-Africa Joint Research Center, Chinese Academy of Sciences, Wuhan 430074, Hubei, China University of Chinese Academy of Sciences Beijing China; 4 East African Herbarium, National Museums of Kenya, P.O. Box 45166 00100, Nairobi, Kenya Sino-Africa Joint Research Center, Chinese Academy of Sciences Wuhan China

**Keywords:** Aberdare ranges, conservation, elevation, species richness, temperature, vascular plants

## Abstract

Distribution patterns of biodiversity and the factors influencing them are important in conservation and management strategies of natural resources. With impending threats from increased human population and global climatic changes, there is an urgent need for a comprehensive understanding of these patterns, more so in species-rich tropical montane ecosystems where little is known about plant diversity and distribution. Vascular species richness along elevation and climatic gradients of Aberdare ranges forest were explored. A total of 1337 species in 137 families, 606 genera, 82 subspecies and 80 varieties were recorded. Correlations, simple linear regression and Partial least square regression analysis were used to assess richness and diversity patterns of total plants, herbs, shrubs, climbers, arboreal and endemic species from 2000–4000 m above sea level. Total plant species richness showed a monotonic declining relationship with elevation with richness maxima at 2000–2100 m a.s.l., while endemic species richness had a positive unimodal increase along elevation with peaks at 3600–3700 m a.s.l. Herbs, shrubs, climbers and arboreal had significant negative relationships with altitude, excluding endemism which showed positive relations. In contrast, both air and soil temperatures had positive relationships with taxa richness groups and negative relations with endemic species. Elevation was found to have higher relative influence on plant richness and distribution in Aberdare ranges forest. For effective conservation and management of biodiversity in Aberdare, localized dynamic conservation interventions are recommended in contrast to broad and static strategies. Establishment of conservation zones and migration corridors are necessary to safeguard biodiversity in line with envisaged global climatic vicissitudes.

## Introduction

Tropical afromontane ecosystems are renowned hotspots of biological diversity often with significant numbers of endemic species (Mittermeier et al. 1998; Lovett et al. 2005; Sosef et al. 2017). A combination of non-random climatic and abiotic gradients, and evolutionary processes in montane forests have been reported to influence the myriad distribution patterns and composition of plants’ communities found along these mountains (Lomolino 2001). Since montane ecosystems are small and isolated from other similar ecosystems, plant communities in these regions face relatively high extinction rates and low immigration rates (Newmark and McNeally 2018). Therefore, to sustainably manage and conserve these ecosystems an authentic conceptual framework (comprehensive understanding) of species richness and distribution patterns is a prerequisite (Lovett et al. 2005; Sosef et al. 2017; Anderson-Teixeira 2018) . According to Dyakov (2010), plant species are distributed in variable habitats but are most abundant in areas which represent their ecological optimum. Also, Lovett (1999) argued that these plants’ composition and distribution patterns reflect the underlying anthropogenic disturbances. Therefore, an understanding of these patterns can be used to prioritize regions that either need immediate or different management interventions so as to conserve the targeted species.

Previous studies have reported a significant relationship between elevation and plant communities, indicating that elevation is a strong predictor of vegetation structure and richness (Lomolino 2001, Berhanu et al. 2017). However, the extent of its influences remains unclear since it has both indirect effect on species richness and direct effects on environmental complexes (i.e., temperature, growing season, precipitation, wind velocity, atmospheric pressure and evaporation) which are also crucial in spatial patterns of plants (Whittaker 1967; Blundo et al. 2012; Dyakov 2014). Thus, the trends observed in spatial patterns on a mountainous ecosystem cannot be explained by a single factor but rather by an interaction of multiple factors (Lee et al. 2013; Trigas et al. 2013). The effects of these environmental factors are dynamic and vary among different plant groups and growth forms (Zhou et al. 2019). Bhattarai and Vetaas (2003) found significant trends between woody life forms and elevation gradient, but none among the herbaceous species of the Himalayas in Nepal. Dissimilar distribution of plant communities in relation to environmental gradients has been described in many studies (Hamilton and Perrott 1981; Lovett 1996; Grytnes and Vetaas 2002; Vetaas and Grytnes 2002; Bhattarai and Vetaas 2003; Schmitt et al. 2010; Lee et al. 2013; Trigas et al. 2013; Berhanu et al. 2017).

The Aberdare ranges present an interesting ecosystem in that the northern part of this forest is almost at the equator and the western slopes form part of the easternmost wall of the Gregory Rift valley (Schmitt 1991; Bennun and Njoroge 1999). It is one of the five major water catchment towers in Kenya with three major rivers emanating from this forest, i.e. the Tana, Athi and Ewaso Ng’iro rivers (Muiruri 1978; KFS 2010). Overall, the Aberdare forest provides invaluable social, economic and environmental benefits with estimates indicating that at least one in three people in Kenya depends in some way on the natural resources from this ecosystem (Ark and Group 2011; Rhino 2016). These ranges are renowned for their geographically diverse taxa and high endemism (Hedberg 1964; Hedberg and Hedberg 1979) due to wide elevational gradient and other biotic and abiotic factors (Schmitt 1991; Lambrechts et al. 2003; KFS 2010). The heterogeneous flora along the altitude breath stands out as unique biota above the warmer plains surrounding these volcanoes (Hedberg and Hedberg 1979). Despite its complex vegetation diversity, little has been done to quantify the species richness, composition and altitudinal turnover in this afromontane forest. This study, therefore, aimed at describing the overall floral richness patterns and identify areas with strikingly unique richness so as to provide a baseline framework for immediate or future localized and effective conservation strategies. The specific objectives of the study were to (a) investigate the distribution patterns of the plants, herbs, shrubs, climbers, arboreal and endemic species in Aberdare ranges, (b) explore the degree of influence of elevation, air and soil temperatures on the richness patterns of the same taxon groups, and (c) evaluate conservation priorities based on observed species richness and distribution patterns for the entire Aberdare ecosystem.

## Materials and methods

### Study site

**Figure 1.**
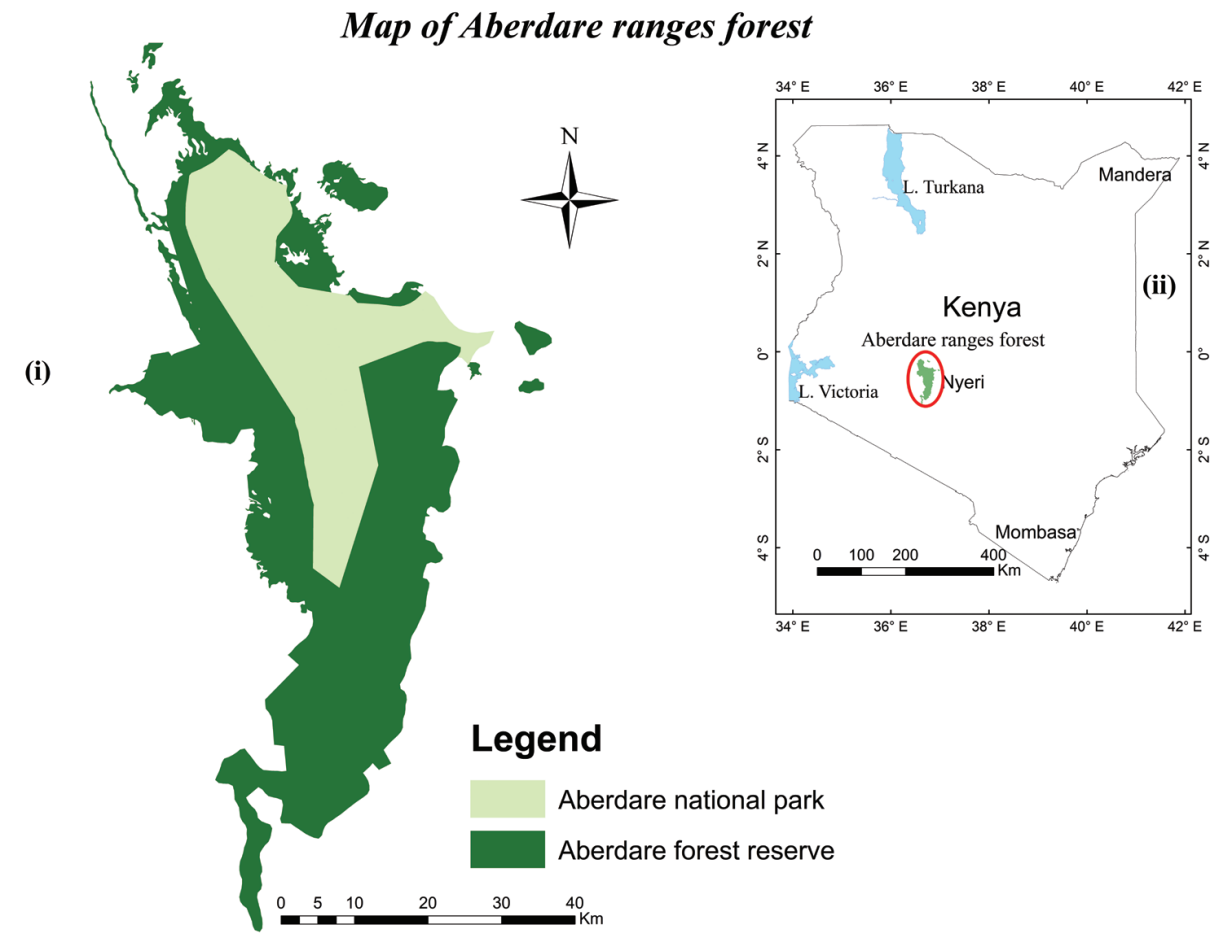
The location of Aberdare ranges forest, (**i**) two sections of forest (**ii**) map of Kenya.

The study was carried out in the Aberdare mountains, located in central parts of Kenya (Bennun and Njoroge 1999). It stretches for 120 km from north to south from latitude 00°08' to 00°42' south, with an expanse of about 40 km across at its widest point between longitude 36°31' to 35°57' east (Butynski 1999) (Fig. 1). The Aberdare ranges are characterized by undulating hills formed through uplift and warping, then later shaped by volcanism and faulting of the earth surface from early Tertiary to the Pleistocene (Peltorinne 2004), giving rise to geographically isolated islands of complex tropical-alpine vegetation (Hedberg 1964). There are two main peaks; Oldonyo Lesatima (4000 m a.s.l.) to the north and Il Kinangop (3906 m a.s.l.) to the south, separated by a long stretch of land above 3000 m elevation (Muiruri 1978; Bennun and Njoroge 1999). Aberdare exhibits a unique topography sloping gradually to the East while, in contrast, the western slopes drop rapidly along imposing fault escarpments towards the Kinangop plateau and finally the Gregory Rift Valley, giving way to a number of torrential waterfalls that cascade into deep u-shaped ravines (KFS 2010; KWS 2010).

Similar to other East African mountains, vegetation in Aberdare is typical afromontane type characterized by heterogeneous vegetation structure along the elevational gradients (Hedberg 1951; Myers et al. 2000). Three broad vegetation zonations have been described by Schmitt (1991) based on floristic compositions, including the montane forest belt at lower altitudes, the subalpine zone at mid elevation, and the alpine zone at the summit. Administratively, this forest is managed in two sections, i.e., Aberdare national park, size *ca.* 76700 ha., managed by Kenya Wildlife Service (KWS), and Aberdare forest reserve, size *ca.* 139500 ha., governed by Kenya Forest Service (KFS). The national park is situated at higher elevations above *ca.* 2600 m a.s.l. and the regions below this park constitute the forest reserve composed of three forest blocks, Aberdare, Kikuyu escarpment, and Kipipiri forest reserve (KWS 2010; KFS 2010, Lambrechts et al. 2003). Altitude is from 1800 m to 4000 m a.s.l. (Bennun and Njoroge 1999). Climate in Aberdare forest is dominated by the passage of the Inter-Tropical Convergence Zone north and south during its annual cycle, producing a bimodal rainfall distribution (CEPF 2012). Long rains usually come from March to May and short rains from October to November (Lambrechts et al. 2003; KFS 2010). Annual rainfall varies with altitude and exposure to the dominant winds from the Indian Ocean, ranging from 1000 mm on the drier north-western slopes to as much as 3000 mm on the south-eastern slopes (Bennun and Njoroge 1999). The mean maximum temperature is 25.8 °C. while mean minimum temperature is 10.3 °C. (KFS 2010; CEPF 2012).

### Data Sources

The floristic surveys were carried out from the year 2016 to 2018, during the optimum flowering and fruiting periods which were mainly after the long and short rains of March-May and November-December respectively. A team of botanists from the National Museums of Kenya and Sino-Africa Joint Research Center undertook the surveys. Fertile voucher plant specimens, with either flower, fruit or both were collected, pressed, and dried. Specimens were identified to species level using varied taxonomic monographs and botanical guide books (FTEA 1952–2012; Blundell 1992; Agnew and Agnew 1994; Beentje et al. 1994; Agnew 2013). Plant species were further categorized into their life forms, including (i) herbs (plants less than 50 cm or less than 100 cm but annual), (ii) shrubs (plants between 50cm to 5 m tall), (iii) climbers (plants with twining herbaceous or woody stems), and (iv) arboreal (plants taller than 5m) (Raunkiaer 1934, Schmitt 1991, Bao et al. 2018). Finally, the dried voucher specimens were deposited in the East African Herbarium (EA), Nairobi, and Wuhan Botanical Garden herbarium (HIB), China.

Endemic species were recorded from published literature and updated by cross-checking with the online occurrence data in Global Biodiversity Information Facility (**GBIF**) (https://www.gbif.org). In addition to field collections, other plant species in Aberdare ranges forest were obtained from the previously collected specimens in the EA herbarium catalogues, series of Flora of Tropical East Africa (FTEA) and other standard references (e.g., Schmitt 1991). Relative altitude range sizes of plant species in Aberdare i.e., the maximum and minimum altitude where a species has been previously recorded or collected, were determined from our collections, as well as from other specimens in the EA herbarium and the standard botanical references. Based on the contemporary description of the Flora of Kenya, information on the floral species and their geographical distributions was considered adequate for this study.

Both air and soil temperature data were obtained from Schmitt (1991), where he calculated the average air temperature from 28 months’ recordings from four locations at different altitudes in Aberdare ranges. Extrapolation was done based on a calculation from East African Meteorological Department (1970) of a decrease of 1.7 °C. for every 1000 feet of altitude equivalent to 0.56 °C. per 100 m elevation. Regarding the soil temperature, Schmitt (1991) obtained the mean of 9 months recordings at 70 cm depth of soil in eight different elevations in the Aberdare ranges. He calculated an average of 0.52 °C. decrease per 100m elevation between 1900−3200 m a.s.l. and 1.5 °C. decrease per 100 m between 3200−3600 m a.s.l. (Braun 1986; Schmitt 1991). This criterion was applied to calculate soil temperatures at altitude bands where there were no recordings.

### Data analysis

The Aberdare mountain rises from 1800−4000 m a.s.l. However, the onset of continuous forest cover differs in elevation at various sites of forest edges. To control the biases of uneven elevation of forest margins and disjunct forest blocks, we analyzed species from 2000−4000 m a.s.l. as this represents relatively the continuous forest cover in the entire ecosystem (Schmitt 1991, Butynski 1999). Elevation gradient was divided into 20 bands of 100 m interval between the 2000−4000 m altitude in the same manner as Vetaas and Grytnes (2002), Bhattarai et al. (2004) and Grau et al. (2007). Recorded plant species were then interpolated at these 20 100 m bands giving an estimate of gamma diversity defined by Lomolino (2001) as total richness of an entire elevation band. The assumption here was that each species was present in all the elevation bands within its altitude range size, ignoring any disjunctions in its distribution along elevation gradient. Interpolation of species facilitates analysis where there is incomplete sampling as in this study. A comparative analysis conducted on empirical and interpolated species richness data of the Baekdudaegan mountains of South Korea showed similar spatial distribution patterns (Lee et al. 2013). Hence interpolation of taxon richness was best suited for our study as it can give reliable results similar to complete sampling. Each plant species, subspecies or varieties were treated as individual taxa. The relationship between all these plant groups and elevation, air temperature and soil temperature were investigated in IBM SPSS statistic 25 software.

Descriptive statistics were explored to interpret data distributions and normality tests (Table 1). Moderate skewness and negative kurtosis were observed indicating nearly normal distributions as they were below 1 and 2.5 respectively (Zhao and Fang 2006). Arboreals were square-root transformed since it failed Kolmogorov-smirnov normality tests (P < 0.023). No transformations were done for other groups as their distributions were normal (P > 0.05). Simple scatter plots were used to observe if non-linear relationship existed between plant groups and combined environmental variables studied. All plant groups showed linear relationship, hence Pearson’s Correlation analysis was done. Multiple regression model was best suited for data analysis, however, there was significant multicollinearity between the predictors (r > 0.07), which would have given unreliable results. Therefore, Partial Least Squares (PLS) regression model was used (Tobias 1995). PLS regression is robust in multicollinear variables and it focuses on components with maximum covariance with dependent variable (Maestre 2004; Davis et al. 2007; Carrascal et al. 2009), hence, an environmental factor with the highest projection on richness patterns of plants groups could be identified through this model. Relative importance of individual predictor was evaluated using Variable Importance in Projection (VIP) values i.e., the higher the VIP the higher the importance in projection. PLS regression with a Python 2.7 extension module was used in IBM SPSS statistic 25 software. In addition to PLS regression, simple linear regression of each environmental factor on each studied plant group was also calculated.

**Table 1.** Summary of descriptive statistics of environmental factors and vascular plants groups in Aberdare ranges forest.

Variables	N	Minimum	Maximum	Mean	SD	Skewness	Kurtosis	K-S (P)
Environmental factors								
Elevation	20	2050	3950	3000	132.288	0	-1.200	0.2
Air temperature	20	3.86	16.58	9.73	4.129	0.393	-1.063	0.2
Soil temperature	20	1.3	18.02	11.47	5.098	-0.602	-0.763	0.2
Species richness								
Total plants	1337	171	1032	593.15	62.952	0.087	-1.401	0.2
Endemic species	63	20	37	28.35	6.124	0.027	-1.737	0.124
Plant Life forms								
Herbs	888	144	691	446.25	191.877	-0.224	-1.443	0.2
Climbers	101	2	83	33.15	28.372	0.424	-1.349	0.095
Shrubs	198	24	151	76.05	41.19	0.572	-0.997	0.135
Arboreal	150	1	116	37.7	38.811	0.996	-0.408	0.023

## Results

**Figure 2.**
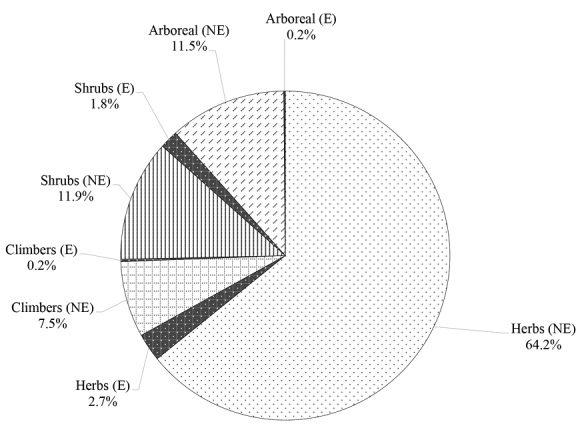
Proportions of endemic and non-endemic plants species life forms. (E – endemics, NE – non endemics).

The Aberdare ranges forest has high plant diversity. The majority of the plants recorded were seed plants totaling 1255 taxa including forbes while 86 were ferns. The top-ranking families as per the number of taxa were Asteraceae 11%, Poaceae 8%, Fabaceae 7%, Lamiaceae 4%, Cyperaceae 4%, Rubiaceae 4%, Euphorbiaceae 3% and Orchidaceae 3%. Other families had fewer species with some having a single species. According to plant life forms, most taxa were herbs 64.2%, then shrubs 11.9%, arboreal 11.5% and climbers were 7.5% of the total taxa recorded (Fig. 2). Endemic species composed of varied life forms were 4.7% of the total taxa recorded. Average altitude range for total taxa excluding endemic species was 1585 m. Among the life forms, herbs had the highest average altitude range at 1614 m, climbers 1578 m, shrubs 1490 m, and the lowest was arboreal at 1350 m. Endemic species had the lowest average altitude range of 1139 m.

Pearson’s correlation analysis showed significant relationship between total plants, endemic species, life forms groups and environmental variables (see Table 2). Elevation had negative correlation with herbs, shrubs, climbers and arboreal. Total plants also had negative correlation with elevation but endemic species showed a weak positive relationship. Both air and soil temperatures showed significant positive relations with all life form groups. Endemic species had negative correlations with air and soil temperature while total plants had positive relationship with both temperatures. Simple linear regression indicated variations in richness patterns of the studied taxa groups. Elevation explained 99.3% of total plant richness and 69.2% for endemic species. Air and soil temperatures also had higher influence on total plant richness explaining 97.4% and 93.2% respectively. Strikingly, temperature variables showed lower prediction on endemic species with just 64.9% explained by air temperature and 58.9% by soil temperatures. In general, all the environmental factors had significant prediction on all life form groups’ richness and distribution patterns (*P* < 0.000) (Table 2). Soil temperature was found to have the least influence on richness patterns prediction compared to other environmental factors.

**Table 2.** Correlation analysis between environmental factors and plant species groups.

Correlations										
		Altitude	Air temp.	Soil temp.	Total plants	Herbs	Shrubs	Climbers	(Sqrt) Arboreal	Ende-mics
Altitude	Pearson Correlation	1	-.985**	-.977**	-.997**	-.991**	-.975**	-.977**	-.984**	.832**
	Sig. (2-tailed)		0.000	0.000	0.000	0.000	0.000	0.000	0.000	0.000
	N		20	20	20	20	20	20	20	20
Air temp.	Pearson Correlation		1	.938**	.987**	.963**	.992**	.982**	.994**	-.806**
	Sig. (2-tailed)			0.000	0.000	0.000	0.000	0.000	0.000	0.000
	N			20	20	20	20	20	20	20
Soil temp.	Pearson Correlation			1	.965**	.984**	.912**	.913**	.932**	-.767**
	Sig. (2-tailed)				0.000	0.000	0.000	0.000	0.000	0.000
	N				20	20	20	20	20	20
Total plants	Pearson Correlation				1	.991**	.981**	.985**	.988**	-.853**
	Sig. (2-tailed)					0.000	0.000	0.000	0.000	0.000
	N					20	20	20	20	20
Herbs	Pearson Correlation					1	.948**	.959**	.961**	-.844**
	Sig. (2-tailed)						0.000	0.000	0.000	0.000
	N						20	20	20	20
Shrubs	Pearson Correlation						1	.989**	.995**	-.825**
	Sig. (2-tailed)							0.000	0.000	0.000
	N							20	20	20
Climbers	Pearson Correlation							1	.988**	-.877**
	Sig. (2-tailed)								0.000	0.000
	N								20	20
(Sqrt) Arboreal	Pearson Correlation								1	-.828**
	Sig. (2-tailed)									0.000
	N									20
Endemics	Pearson Correlation									1

**. Correlation is significant at the 0.01 level (2-tailed). (Sprt) refers to transformed variable by square rooting

Total plants showed significant monotonic declining trend with increasing elevation (β = − 0.997, *R^2^* = 0.95). Total plants species richness peaked at 2000−2100 m a.s.l. with 1032 taxa then gradually declined to just 171 taxa at the summit of the mountain. On the contrary, endemic species increased with increasing elevation (β = 0.832, *R^2^* = 0.692) with richness maximum at 3300−3700 m a.s.l., suggesting that endemism was favored in high altitudes with associated harsh climatic conditions. Similar significant monotonic declining trends along the altitude were also observed among the life forms groups; herbs (β = − 0.991, *R^2^* = 0.982), shrubs (β = − 0.975, *R^2^* = 0.950), climbers (β = − 0.977, *R^2^* = 0.954), and aboreals (β = − 984, *R^2^* = 0.983). Trends of life form groups along air and soil temperature gradients are listed in Table 3.

**Table 3.** Simple linear regression of altitude, air temperature, soil temperature and each plant group.

Environmental variables	Total plants	Endemics	Herbs	Shrubs	Climbers	Arboreal
Altitude						
Pearson Correlation (r)	-0.997	0.832	-0.991	-0.975	-0.977	-0.984
Sig (1-tailed) (p < 0.05)	0.000	0.000	0.000	0.000	0.000	0.000
Model Summary (R^2^)	0.993	0.692	0.982	0.950	0.954	0.969
Air Temperature						
Pearson Correlation (r)	0.987	-0.806	0.963	0.992	0.982	0.994
Sig (1-tailed) (p < 0.05)	0.000	0.000	0.000	0.000	0.000	0.000
Model Summary (R^2^)	0.974	0.649	0.928	0.984	0.964	0.988
Soil Temperature						
Pearson Correlation (r)	0.965	-0.767	0.984	0.912	0.913	0.932
Sig (1-tailed) (p < 0.05)	0.000	0.000	0.000	0.000	0.000	0.000
Model Sum (R^2^)	0.932	0.589	0.968	0.832	0.833	0.869

PLS regression model indicated the importance of each studied environmental factor in projecting richness patterns among the studied groups. Elevation, air and soil temperatures projected significant richness trends for total plants (*R^2^* = 0.988) and low projection in endemic richness trends (*R^2^* = 0.639). Other proportions of variance explained by our PLS model in herbs, shrubs, climbers and arboreal were also high and significant (Table 4). Elevation was indicated to have the highest relative importance in richness patterns of all the plant groups (V.I.P. > 1) (Maestre 2004). Air temperature showed high relative importance in projection of all studied groups except for the herbs which was slightly below 1 (Table 4). Soil temperature was the least influencing variable overall in all the studied groups. Elevation had negative and positive relationship with total plants and endemic species respectively. In contrast, air and soil temperatures showed positive relationships with total plants and life form groups but negative relations with endemic species It was interesting to note that similar results shown in correlation and simple linear regression analysis were also observed in PLS model.

**Table 4.** Partial least square regression of combined environmental factors and species richness groups.

**Species Groups**	**Parameters**	**V.I. P**	**W**	**L**	**Proportion of Variances explained (adjusted R^2^)**
Total Plants					0.988
Elevation	-0.553	1.014	-0.585	-0.583	
Air Temperature	3.423	1.004	0.580	0.575	
Soil Temperature	-9.427	0.982	0.567	0.574	
Endemic species					0.639
Elevation	0.053	1.037	0.599	0.583	
Air Temperature	3.241	1.005	-0.580	-0.576	
Soil Temperature	2.615	0.957	-0.552	-0.574	
Herbs					0.980
Elevation	-0.316	1.012	-0.584	-0.583	
Air Temperature	-10.046	0.984	0.568	0.575	
Soil Temperature	8.803	1.005	0.580	0.574	
Shrubs					0.941
Elevation	-0.066	1.015	-0.586	-0.583	
Air Temperature	5.936	1.033	0.596	0.576	
Soil Temperature	-4.647	0.95	0.548	0.574	
Climbers					0.935
Elevation	-0.098	1.020	-0.589	-0.583	
Air Temperature	-0.743	1.025	0.592	0.576	
Soil Temperature	-5.419	0.953	0.550	0.574	
Arboreals					0.962
Elevation	-0.005	1.014	-0.586	-0.583	
Air Temperature	0.392	1.024	0.591	0.576	
Soil Temperature	-0.241	0.960	0.555	0.574	

### Plants of special concern

Aberdare ranges forest harbors a number of globally important plant species. A total of 73 species have been assessed globally to be threatened and 30 species are possibly threatened according to Botanic Gardens Conservation International threat search 2019 (https://tools.bgci.org/threat_search.php). This is a clear indication of the global importance of Aberdare ranges forest as a biodiversity hotspot and the urgent need for effective conservation measures to protect the threatened species and the rich plant diversity in general. The majority of the threatened species were herbs with 70 species, shrubs 12, climbers 11 and the arboreal numbered 10 (see Appendix 1).

## Discussion

### Plant species richness and distribution patterns

Plants diversity in Aberdare ranges is high, based on the total taxa recorded in our study. This was higher than the previous survey done by Schmitt (1991) which documented 778 species in 128 families and 421 genera. Altitude was found to have relatively higher influence on distribution and richness patterns of plant species in this forest. This suggests that the heterogeneous vegetation structure exhibited in the Aberdare ranges is a manifestation of altitude increase. This phenomenon has attracted a lot of debate with several factors mentioned as explanatories for habitat heterogeneity, e.g., energy, water, soils, and area. However, these factors are known to have direct influence on plant physiological processes which in turn control plant growth and spatial richness; at the same time these factors are directly influenced by altitude (Whittaker 1967; Rahbek 1997; Schmitt et al. 2013; Berhanu et al. 2017). Therefore, altitudinal gradient can be regarded as an overall fundamental factor with indirect effects on species richness patterns. Dyakov (2014) argued that altitude can be used to provide insightful information on plants richness and distribution patterns, especially in ecosystems where other abiotic parameters are missing and this notion has been supported by this study. Total plant species in Aberdare showed a monotonic declining relationship with optimum altitude ranging between 2000–2100 m a.s.l., which was simply the foot of the Aberdare ranges. The aggregation of species at lower elevation indicated mass effect which coincided with Rahbek’s (1997) observation that lowlands represent sink habitats with higher species richness than other higher elevation zones. This higher richness is likely due to the infusion of species from surrounding lowland vegetation which could not survive at higher elevations (Rahbek 1997). It is also probable that human transferred species might have been perfectly integrated and naturalized during early historic community settlements in and around Aberdare mountains (Trigas et al. 2013). The known harsh environmental constraints at higher elevations such as cooler temperatures, low energy, shorter growing seasons, solifluction, isolation, etc., could have deterred to an extent any immigration, dispersal and invasiveness of new plant species at these elevated areas (Hedberg 1964; Vetaas and Grytnes 2002; Dyakov 2014). Furthermore, monotonic decline of species richness could be due to the absence of true montane flora in Aberdare, where the current richness patterns are a result of the spread of lowland species that could withstand and adapt to the mentioned harsh conditions associated with higher elevations (Hedberg and Hedberg 1979; Trigas et al. 2013). Numerous studies in similar montane ecosystems have reported monotonic decline of floristic richness. In Ethiopian afromontane vegetation, species richness peaked between 1600–1700 m a.s.l. which was lower than Aberdare despite both mountains flanking the Great Rift Valley (Berhanu et al. 2017). Vetaas and Grytnes (2002), using interpolated data of Nepal Himalayas, found a hump-shaped unimodal relationship with maxima species richness between 1500–2500 m a.s.l., which was similar to this study.

There was a slight increase in richness of herbs between 2000–2100 m a.s.l. then a monotonic decline as altitude increases. Herbs showed higher gamma diversity i.e., total species in each altitude band, compared to other studied plant groups in entire altitude gradient of Aberdare ranges. Regarding this observation, this study contrasted with Schmitt et al. (2010) generalization that herbs are more sensitive to small-scale changes in environmental conditions while woody plants are affected by environmental changes at larger scale. If this was the case then woody species richness i.e., shrubs and arboreal would be more abundant at higher altitudes which was not observed in this study. As floral richness is known to be maximum at altitudes associated with their optimal climatic gradients (Lomolino 2001; Dyakov 2014), based on this study 2000–2200 m a.s.l. seems to be the focal altitude for total plants in Aberdare ranges forest. The effects of heavy deforestation at lower altitudes in close proximity to local communities around the forest (Butynski 1999; Lambrechts et al. 2003; KWS 2010; KFS 2010) were not shown in our study. It was expected that richness in shrubs and arboreal would be low at the foot of the range as a result of overexploitation then a slow or even absent succession by arboreal species afterwards.

### Endemism

A total of 63 species were endemic in Aberdare ranges forest. Most of these endemics were herbs - with 35 species, then shrubs 23, climbers 3 and the lowest were arboreal with 2 species (see Appendix 2). The altitudinal range sizes, i.e., between the minimum and maximum altitude, of these endemic species varied from as low as 100 m for *Cissampelos friesiorum* Diels, 150 m for *Senecio margaritae* C.Jeffrey to 2500 m for *Adenocarpus mannii* (Hook.f.) Hook.f. and *Erica sylvatica* (Welw. ex Engl.) Beentje. Endemic species had positive relations with elevation and negative relations with both air and soil temperatures. There was a continuous spread of endemic species in the entire forest; however, higher richness was observed at higher altitude. Endemism richness maxima ranged between 3200–3900 m a.s.l., which overlapped with the endemic species of the Himalayas mountain which peaked between 3500–4500 m a.s.l. (Vetaas and Grytnes 2002). This study supported the generalization that endemic species as restricted range taxa were manifested by the survival of species in refugia during Pleistocene climatic fluctuations and/or their resilience and adaptation to unique long-term abiotic conditions that promoted morphological differentiation of relict taxa (Lovett and Friis 1994; Lovett et al. 2000). Higher endemism towards the top of Aberdare ranges might be a result of temporal individual responses of plant species to climatic vicissitudes leading to novel assemblages at high altitudes as different species move individually up the slope (Lovett et al. 2005). There was a notably rapid increase in endemic richness at 2700–3000 m a.s.l. which coincided also with the rapid decrease in total plant species richness. This striking feature was also observed among the endemics of the Himalayas in Nepal although at higher altitudes above 4000 m a.s.l. (Vetaas and Grytnes 2002). The authors at Himalayas Nepal argued that timberline and glaciation limits are responsible for the inverse relationship between endemism and total plant richness. However, this view was only partially supported in our study due to the current absence of glaciers in the Aberdare ranges which are thought to have disappeared during early Holocene glaciation (Young 1980; Rosqvist 1990; Mark and Osmaston 2008). This study therefore suggests that perhaps localized competition among plant species has an effect on endemic richness and diversity although this largely remains a speculative concept. Increasing isolation and decreasing surface area at higher elevation which are responsible for fragmentation of species population, have been argued to facilitate endemism because of the small population’s vulnerability to speciation (Trigas et al. 2013 and references there in). The formation of the Aberdare mountains during the break-up of Gondwanaland millions of years ago led to isolation of plant species that existed then. Over time these species have undergone speciation, resulting in numerous endemic species (Butynski 1999).

### Conservation priorities

In the wake of elevated anthropogenic threats and global climatic vicissitudes, conservation and management of Afromontane ecosystems should be prioritized, particularly for future biodiversity and sustainable provision of ecological services (Muiruri 1978; Myers et al. 2000; Hannah et al. 2002; Kiringe et al. 2007; KFS 2010; Schmitt et al. 2010; Di Minin et al. 2013; Anderson-Teixeira 2018). Afromontane forests are thought to provide survival options for plants in a changing climate through the close physical proximity of a wide range of habitats with varied biotic and abiotic factors (Lovett et al. 2005). Thus, the Aberdare ranges with its proven diverse flora and unique physical properties calls for more effective management interventions. Floristic composition and spatial patterns studied in this forest can steer conservation strategies by pinpointing exceptional rich species and ecologically unique zones (Platts et al. 2010). Thence, the type of management regime to be implemented should target the causal factors of observed richness patterns since species richness is an indicator of environment history (Lovett et al. 2000; Platts et al. 2010). This implies that any conservation intervention to be undertaken should focus on maintaining the forms and magnitudes of disturbances the existent vegetation has adapted to. For the case in Aberdare, higher endemic richness at higher elevations indicates the existence of undisturbed long-term environmental stability as argued by Lovett and Friis (1994), Sosef (1994) and Lovett et al. (2000). Therefore, disturbances should be minimized at this zone because the vegetation might not be resilient to perturbation. Networks of roads and camp sites should be reduced, if not totally eliminated, in upper parts of the forest (KFS 2010; KWS 2010). According to Connell (1978), Phillips et al. (1994) and Lovett et al. (2000) high species diversity is a result of intermediate levels of disturbance that enhances recruitment and regeneration of new species. If this concept of disturbance-driven species richness is applied at lower elevations of Aberdare, where there is a history of exploitation by local community (Butynski 1999), then it means that the great species richness observed at this region could be due to disturbance. Therefore, a suitable management intervention should complement the previous disturbances at appropriate levels and magnitude so as to maintain plant species complementation. This will necessitate some controlled small-scale extraction and utilization of forest resources around the foot of Aberdare ranges. Regulated extraction and utilization of forest products could be the best conservation strategy at species level as this can be an intermediate disturbance if well managed. The carrying capacity of the vegetation area to be grazed should be established prior to grazing.

In view of envisaged climatic changes, it has been suggested that ecosystems migrate to new regions as a result of climatic fluctuations (Hannah et al. 2002; Lovett et al. 2005). In fragmented ecosystems characterized by isolated habitats that are surrounded by agricultural or other human activities, such ecosystems will have no room for migration resulting in total collapse (Hannah et al. 2002). This scenario resonates with the present situation in the Aberdare ranges with agricultural activities surrounding the forest almost entirely. To overcome the impending predicament of ecosystem collapse, creation of corridors seems to be the best strategy, perhaps the only way, to facilitate ecosystem migration and enhance survival of species (Lovett et al. 2005).

Completion of a perimeter electric fence around the Aberdare ranges almost a decade ago has impacted significantly on both ecological and economic aspects of this region (Ark and Group 2011). However, the long-term effects of this fence regarding migration and population increase of wildlife, particularly the large herbivores, have not been adequately addressed (Butynski 1999; Ark and Group 2011). A complete barrier to the migration of elephants has increased damage to vegetation mostly around their traditional migratory routes to the lower Laikipia savanna and Mount Kenya (Ark and Group 2011). Prolonged damage to vegetation in such areas will result in larger open grounds with reduced plant diversity which will lead to minimal utilization of nutrients by plants since the full range of plant niches is not covered (Lovett et al. 2005). This unbalanced destruction of vegetation in the Aberdare ranges will disrupt its ecological equilibrium and threaten the ecosystem functioning and sustainability in the long run (Tilman et al. 1996, 2006). In addition, the expanding populations of range-restricted elephants and other herbivores will increase vegetation damage in the near future. A management intervention in this respect should be at vegetation level and must aim at balancing utilization and regeneration of resources to maintain adequate vegetation cover and rich species diversity, thus maintaining ecological balance and ecosystem productivity of the Aberdare range forest. Migratory corridors should be established, perhaps along the traditional migratory routes, to facilitate seasonal migration of large herbivores in an out of Aberdare ranges.

Wildfires in the Aberdare ecosystem have been common incidences for past decades, occurring mostly during dry seasons in the months of January, February or March and a few cases in September (KFS 2010; KWS 2010). Causes of these fires vary from arsonist ferrying poles, charcoal burning, nearby litter burning during farmland preparations, camp fires and improper cigarette butt disposal by tourists (KFS 2010; KWS 2010; Njeri et al. 2018). Vegetation damages caused by fire are higher at the upper parts of Aberdare including the established plantations due to higher fuel biomass (Njeri et al. 2018). As a result, fragile habitats like the moorland which harbors high endemic and threatened species are negatively impacted. However, fires have also been found to facilitate regeneration of *Juniperus procera* Hochst. ex Endl., *Bambusa vulgaris* Schrad, *Hagenia abyssinica* (Bruce ex Steud.) J.F.Gmel. including some Pines, Cypress and Eucalyptus trees (KFS 2010; KWS 2010; Nyongesa and Vacik 2018). Therefore, the current management approach of prevention and suppression of fire implemented by the KWS and KFS in Aberdare is feasible in the moorland but misplaced in areas with fire-dependent plant species. This study recommends the implementation of an Integrated Fire Management (IFM) framework proposed by Nyongesa and Vacik (2018) in Mount Kenya forest as they are similar ecosystems. In IFM framework, fire-sensitive sites are protected while prescribed-burning is undertaken in fire-dependent ecosystems.

## Conclusion

Aberdare ranges forest is exceptionally rich with diverse flora and high endemic species. Floral richness in the entire mountain monotically declined along elevation and temperature gradients. Similar declines of richness were also depicted by the plant life form groups suggesting that growth forms can serve as surrogates for spatial physiognomy of plants that can guide in prioritizing specific areas for conservation (Acharya et al. 2011). Endemic species, by contrast, increased along the studied environmental gradients with richness maxima at higher elevations. The sharp increase of endemism at mid elevations coinciding with rapid reduction in total plant richness raises doubt on the role of competition in the evolution of endemic novelties.

In summary, the Aberdare ranges forest is composed of strikingly diverse flora uniquely distributed along elevational and temperature gradients. Observed richness and distribution patterns in the entire Aberdare range can provide tentative estimates for conservation importance (Acharya et al. 2011). For effective conservation and management of this ecosystem, both natural and human-induced changes should be put into consideration, and a dynamic site-specific management intervention be implemented rather than broad static interventions. This study proposes subdivision of the Aberdare ranges forest into conservation zones where different management programs can be implemented at specific zones based on the characteristics and composition of vegetation in those zones. Further, the study recommends enrichment planting with native species along with exotic species in attempts to rehabilitate heavily deforested patches in the Aberdare ranges forest. Exotic species including *Cupressus lusitanica* Mill, *Pinus patula* Schiede ex Schltdl. & Cham. and *Juniperus procera* Hochst. ex Endl. have been planted to about 35, 444 ha without enrichment planting (Butynski 1999). It has been found that the establishment of exclusively exotic species does not accelerate natural succession in degraded lands due to absence of native species (Mosandl and Günter 2008). Therefore, subsequent enrichment planting with native species after plantations of exotic species will likely accelerate restoration of biodiversity especially if animal-dispersed species are planted in Aberdare ranges forest (Mosandl and Günter 2008).

## Appendix 1

Plants of special concern in Aberdare ranges forest

**Table:**

Family	Plant species	Conservation status	Life form	Altitude range (m a.s.l.)
Acanthaceae	*Asystasia lorata* Ensermu Kelbessa	threatened	herb	1400−2000
Amaranthaceae	*Chenopodium murale* L.	threatened	herb	1070−2750
	*Chenopodium opulifolium* Schrad.U	threatened	herb	760−2100
Anacardiaceae	*Rhus longipes* Engl.	threatened	shrub or tree	1000−2400
Apiaceae	*Torilis arvensis* Link	possibly threatened	herb	1560−2850
Apocynaceae	*Carissa spinarum* L	possibly threatened	shrub	0−2250
	*Cynanchum viminale* (L.) Bassi	threatened	herbaceous climber	100−2200
	Pentarrhinum abyssinicum subsp. angolense (N.E.Br.) S.Liede & A.Nicholas	threatened	herbaceous climber	1400−2200
Araliaceae	*Polyscias kikuyuensis* Summerh.	possibly threatened	tree	1750−2620
Asparagaceae	*Asparagus racemosus* Willd.	near threatened	woody climber	1160−2900
Aspleniaceae	*Asplenium adamsii* Alston	possibly threatened	herb	2400−3400
	*Asplenium aethiopicum* (Burm.f.) Becherer	threatened	epiphyte	1150−3700
	*Asplenium monanthes* L.	threatened	herb	1950−3400
	*Asplenium rutifolium* (P. J. Berg.) Kunze	threatened	epiphyte	750−2300
Basellaceae	*Basella alba* L.	threatened	herbaceous climber	0−2450
Bignoniaceae	*Jacaranda mimosifolia* D. Don	threatened	tree	1970−1970
Boraginaceae	*Cynoglossum lanceolatum* Forskál	threatened	herb	1100−3220
	*Heliotropium zeylanicum* (Burm. fil.) Lam.	threatened	herb	0−1740
Brassicaceae	*Barbarea intermedia* Boreau	threatened	herb	3050−3950
	*Cardamine africana* L.	threatened	herb	1000−3400
	*Farsetia stenoptera* Hochst.	threatened	herb	500−1700
	*Thlaspi alliaceum* L.	threatened	herb	3050−3600
Campanulaceae	*Lobelia bambuseti* R.E.Fr. & T.C.E.Fr.	possibly threatened	shrub	2350−4000
Canellaceae	*Warburgia ugandensis* Sprague	threatened	tree	1100−2230
Cannabaceae	*Celtis africana* Burm. fil.	threatened	tree	30−2400
Caprifoliaceae	*Valerianella microcarpa* Loisel.	possibly threatened	herb	2800−3500
Caryophyllaceae	*Corrigiola litoralis* L.	threatened	herb	1200−2190
	*Drymaria cordata* (L.) Roem. & Schult.	possibly threatened	herb	870−2700
	*Uebelinia crassifolia* T. C. E. Fries	possibly threatened	herb	2500−4000
Celastraceae	*Hippocratea goetzei* Loes.	threatened	climber	0−3000
Asteraceae	*Ethulia scheffleri* S.Moore	threatened	herb or subshrub	1500−2500
	*Gynura campanulata* C.Jeffrey	threatened	herb	1600−1615
Asteraceae	*Helichrysum ellipticifolium* Moeser	threatened	herb or subshrub	2500−4800
	*Hypochaeris glabra* L.	threatened	herb	1850−3000
	*Lactuca inermis* Forssk.	possibly threatened	herb	500−3300
	*Laphangium luteoalbum* (L.) N.N.Tzvel.	threatened	herb	300−3850
	*Microglossa pyrifolia* (Lam.) O. Kuntze	possibly threatened	shrub	50−2650
	*Senecio amplificatus* C.Jeffrey	threatened	herb	2900−3500
	*Solanecio angulatus* (Vahl) C. Jeffrey	threatened	herbaceous climber	1800−2500
Convolvulaceae	*Cuscuta australis* Hook.fil.	threatened	herbaceous climber	1750−2170
	*Ipomoea wightii* (Wall.) Choisy	threatened	herb	1040−2400
Cucurbitaceae	*Peponium vogelii* (Hook. fil.) Engl.	threatened	herbaceous climber	10−2600
Cupressaceae	*Cupressus lusitanica* Mill.	possibly threatened	tree	2600−2640
Cyperaceae	Carex monostachya A.Rich.	threatened	herb	2700−4500
	Carex phragmitoides Kük.	threatened	herb	2500−3100
	*Carex vallis-rosetto* K.Schum.	threatened	herb	1000−3300
	*Cyperus afroalpinus* Lye	possibly threatened	herb	1000−3000
	Fimbristylis complanata subsp. keniaeensis (Kük.) Lye	possibly threatened	herb	1500−2700
	*Fimbristylis ovata* (Burm.f.) J.Kern	possibly threatened	herb	0−2200
Dennstaedtiaceae	*Hypolepis goetzei* Hieron. ex Reimers	threatened	herb	2100−3050
Dichapetalaceae	*Dichapetalum madagascariense* (Dup.-Thou.) Poir.	near threatened	climber	1500−2400
Dryopteridaceae	*Arachniodes webbiana* (A.Braun) Schelpe	threatened	herb	1380−2600
	*Dryopteris antarctica* (Baker) C.Chr.	threatened	herb	2500−3320
Ebenaceae	*Diospyros abyssinica* (Hiern) F.White	possibly threatened	tree	0−2400
Euphorbiaceae	*Croton alienus* Pax	threatened	shrub or small tree	1525−1825
	*Euphorbia brevitorta* P.R.O.Bally	threatened	herb	1500−2000
Lamiaceae	*Plectranthus caespitosus* Lukhoba & A.J.Paton	possibly threatened	herb	1500−2850
	Plectranthus punctatus subsp. edulis (Vatke) A.J.Paton	threatened	herb	1800−3200
Fabaceae	Crotalaria agatiflora subsp. engleri (Baker f.) Polhill	possibly threatened	herb	1500−3500
	*Crotalaria jacksonii* Baker f.	threatened	shrubs	2200−3000
	*Lotus corniculatus* L.	possibly threatened	herb	1400−2700
	*Rhynchosia hirta* (Andrews) Meikle & Verdc.	Possibly threatened	herb	0−1850
Lentibulariaceae	*Utricularia gibba* L	threatened	herb	10−2550
Malvaceae	*Hibiscus surattensis* L.	threatened	herb	0−1700
	*Malva verticillata* L.	threatened	herb	1200−4050
	*Sparrmannia ricinocarpa* (Eckl. & Zeyh.) O.Kuntze	threatened	shrub	1550−3380
Myrtaceae	Eucalyptus globulus subsp. maidenii (F.Müll.) Kirkpatrick	threatened	tree	cultivated
Ophioglossaceae	*Ophioglossum vulgatum* L.	possibly threatened	herb	1000−3250
Orchidaceae	*Calanthe sylvatica* (Thouars) Lindl.	threatened	herb	900−3000
	*Cyrtorchis arcuata* (Lindl.) Schltr.	possibly threatened	epiphyte	0−3300
	*Habenaria keniensis* Summerh.	threatened	herb	1950−2950
	Polystachya caespitifica subsp. latilabris (Summerh.) P.J.Cribb & Podz.	threatened	herb	1800−2200
Orobanchaceae	*Phelipanche ramosa* (L.) Pomel	threatened	herb	1735−2250
Passifloraceae	Adenia globosa subsp. pseudoglobosa (Verdc.) de Wilde	threatened	woody climber	0−1850
Pinaceae	*Pinus radiata* D.Don	threatened	tree	2800−2800
Poaceae	*Aira caryophyllea* L.	threatened	herb	2000−4500
	*Andropogon distachyos* L.	threatened	herb	1700−3000
	*Bromus catharticus* Vahl	threatened	herb	2300−2700
	*Calamagrostis epigejos* (L.) Roth	threatened	herb	2000−3000
	*Chloris virgata* Sw.	threatened	herb	10−2120
	*Lolium temulentum* L.	threatened	herb	1900−2300
	*Streblochaete longiarista* (A.Rich.) Pilg.	threatened	herb	1500−3280
Polygonaceae	*Persicaria decipiens* (R. Br.) K. L. Wilson	threatened	herb	1100−1100
	*Grammitis cryptophlebia* (Baker) Copel.	threatened	epiphyte	1900−2150
	*Pleopeltis macrocarpa* (Bory ex Willd.) Kaulf.	possibly threatened	herb	1000−3600
Potamogetonaceae	*Potamogeton pusillus* L.	threatened	herb	600−2000
Pteridaceae	*Pellaea viridis* (Forsk.) Prantl	possibly threatened	herb	650−2250
Rosaceae	*Alchemilla fischeri* Engl.	threatened	herb	2320−3440
	*Prunus africana* (Hook.fil.) Kalkm.	threatened	tree	1350−2750
	*Rubus keniensis* Standl.	possibly threatened	shrubs	1950−2800
Rubiaceae	*Galium spurium* L.	threatened	herb	1250−2700
	*Mussaenda microdonta* Wernham	threatened	shrub or tree	1830−2100
	*Rubia cordifolia* L.	threatened	climber herb	1140−3120
Rutaceae	*Toddalia asiatica* (L.) Lam.	possibly threatened	shrub	0−3000
Scrophulariaceae	*Cycnium tubulosum* Engl.	threatened	herb	130−2400
Smilacaceae	*Smilax aspera* L.	threatened	shrub	1450−2745
Thelypteridaceae	*Christella dentata* (Forssk.) Brownsey & Jermy	possibly threatened	herb	45−2200
	*Stegnogramma pozoi* (Lagasca) K.Iwats.	threatened	herb	2050−3350
	Stegnogramma pozoi var. petiolata (Ching) W.A.Sledge	threatened	herb	2050−3350
Urticaceae	*Obetia radula* (Baker) B.D.Jacks.	threatened	tree	700−2000
	*Parietaria debilis* G.Forst.	possibly threatened	herb	1700−4200
Verbenaceae	*Lantana viburnoides* Vahl	possibly threatened	Woody herb or shrub	0−1950
Xanthorrhoeaceae	*Aloe nyeriensis* Christian	threatened	shrub	1760−2100

## Appendix 2

Endemic species in Aberdare ranges forest

**Table:**

Family	Plant species	Habit
Acanthaceae	*Asystasia lorata* Ensermu Kelbessa	perennial herb
Apiaceae	*Pimpinella keniensis* C. Norman	perennial herb
	Afrosciadium friesiorum var. bipinnatum (C.C. Towns.) P.J.D. Winter	perennial herb
	*Heracleum taylori* C. Norman	perennial herb
Apocynaceae	*Brachystelma keniense* Schweinf.	perennial herb
Asteraceae	Helichrysum formosissimum var. guilelmii (Engl.) Mesfin Tadesse	perennial woody herb or shrub
	*Helichrysum formosissimum* Sch.Bip.	perennial woody herb or shrub
	*Senecio aequinoctialis* R.E.Fr.	perennial woody herb
Asteraceae	*Helichrysum brownei* S. Moore	perennial herb or shrublet
	*Senecio roseiflorus* R.E. Fr.	perennial woody herb or shrub
	*Dendrosenecio battiscombei* (R.E.Fr. & T.C.E.Fr.) E.B. Knox	perennial shrub
	*Helichrysum chionoides* Philipson	perennial shrub
	*Dendrosenecio brassiciformis* (R.E.Fr. & T.C.E.Fr.) Mabb.	perennial shrub
	*Dendrosenecio keniensis* (Baker f.) Mabb.	perennial shrub
	*Senecio jacksonii* S. Moore	perennial herb
	*Dendrosenecio keniodendron* (R.E.Fr. & T.C.E.Fr.) B. Nord.	perennial shrub
	*Carduus silvarum* R.E.Fr.	perennial herb
	*Senecio amplificatus* C. Jeffrey	perennial herb
	*Carduus millefolius* R.E. Fr.	annual or perennial herb
	*Helichrysum gloria-dei* Chiov.	perennial shrub
	*Senecio margaritae* C. Jeffrey	perennial shrub
	*Gynura campanulata* C. Jeffrey	perennial herb
Brassicaceae	*Oreophyton falcatum* O.E. Schulz	perennial herb
Campanulaceae	*Wahlenbergia pusilla* Hochst. ex A. Rich.	perennial herb
	*Lobelia bambuseti* R.E.Fr. & T.C.E.Fr.	perennial shrub
	*Wahlenbergia virgata* Engl.	perennial herb
	*Lobelia telekii* Schweinf.	perennial shrub
	*Lobelia deckenii* (Asch.) Hemsl.	perennial shrub
	Lobelia gregoriana subsp. sattimae (R.E.Fr. & T.C.E.Fr.) E.B. Knox	perennial shrub
Caryophyllaceae	*Uebelinia crassifolia* T. C. E. Fries	annual herb
Cucurbitaceae	*Zehneria subcoriaceae* Y.D. Zhou & Q.F. Wang	perennial herbaceous climber
Cyperaceae	Carex runssoroensis var. aberdarensis Kük.	perennial herb
Ericaceae	*Erica silvatica* (Engl.) Beentje	perennial shrub
	*Erica filago* (Alm & T.C.E.Fr.) Beentje	pluriannual shrub
Fabaceae	*Adenocarpus mannii* Hook.f.	perennial shrub
	*Trifolium cryptopodium* Steud. ex A. Rich.	perennial herb
	*Crotalaria jacksonii* Baker f.	annual shrub
Loranthaceae	Agelanthus sansibarensis subsp. montanus R. M. Polhill & D.	perennial shrub
Lythraceae	Nesaea kilimandscharica var. ngongensis B. Verdcourt	perennial woody herb or shrub
Malvaceae	Abutilon longicuspe var. pilosicalyx Verdc.	perennial shrub
Menispermaceae	*Cissampelos friesiorum* Diels	perennial herbaceous climber
Moraceae	*Dorstenia afromontana* R. E. Fries	annual herb
Passifloraceae	Adenia globosa subsp. pseudoglobosa (Verdc.) de Wilde	perennial shrubby climber
Poaceae	*Eragrostis amanda* Clayton	perennial herb
Primulaceae	*Embelia keniensis* R.E.Fr.	pluriannual arboreal
Ranunculaceae	*Delphinium macrocentrun* Oliv.	perennial herb
	*Anemone thomsonii* Oliv.	perennial herb
	*Ranunculus aberdaricus* Ulbr.	perennial herb
Rosaceae	*Alchemilla johnstonii* Oliver	perennial shrub
	*Alchemilla ellenbeckii* Engl.	perennial herb
	*Alchemilla cyclophylla* T.C.E.Fr.	perennial herb
	*Rubus friesiorum* Gust	perennial shrub
	*Alchemilla argyrophylla* T.C.E.Fr.	perennial shrub
Rubiaceae	*Galium ruwenzoriense* (Cortesi) Ehrend.	perennial herb
	Pavetta abyssinica var. lamurensis Verdc.	pluriannual arboreal
	*Oldenlandia friesiorum* Bremek.	perennial herb
	Canthium oligocarpum subsp. friesiorum (Robyns) Bridson	pluriannual shrub or tree
	Galium glaciale var. satimmae Verdc.	perennial herb
Scrophulariaceae	*Bartsia longiflora* Hochst. ex Benth.	perennial herb
Solanaceae	*Solanum agnewiorum* Voronts.	perennial shrub
Xanthorrhoeaceae	*Aloe nyeriensis* Christian	perennial shrub
Apiaceae	*Afrosciadium friesiorum* (H. Wolff) Winter	perennial herb
	*Afrosciadium englerianum* H. Wolff) P.J.D. Winter	perennial herb
